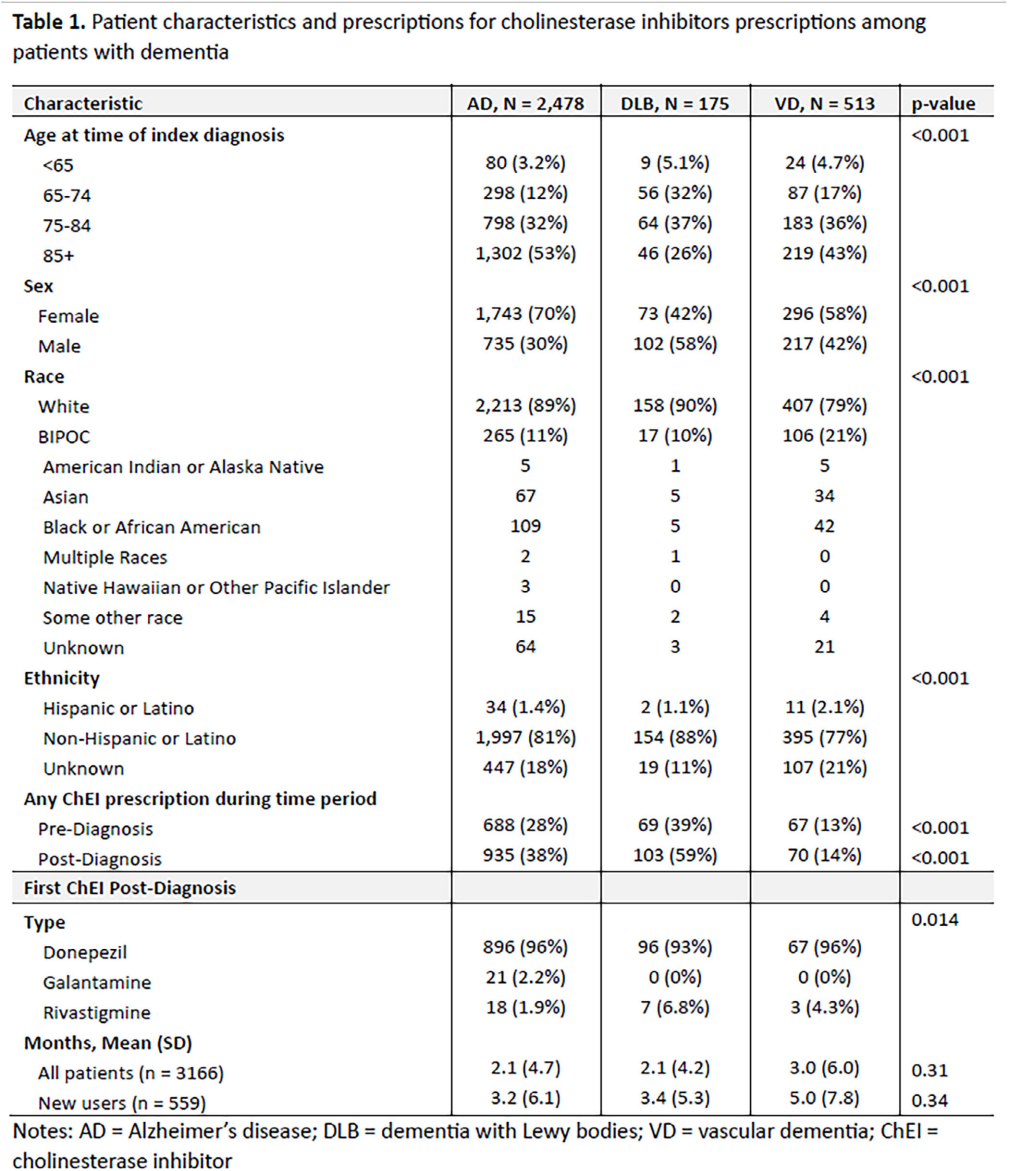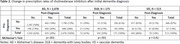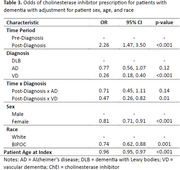# Cholinesterase inhibitors for patients with dementia: Patterns of prescribing and disparities in treatment

**DOI:** 10.1002/alz.091493

**Published:** 2025-01-09

**Authors:** Ahmed Negida, Matthew J Barrett, Kathryn A Wyman‐Chick, Ella A Chrenka, Ece Bayram, Joseph PM Kane, Ann M Werner, Rebecca C Rossom

**Affiliations:** ^1^ Virginia Commonwealth University, Richmond, VA USA; ^2^ HealthPartners Department of Neurology; Center for Memory and Aging, St. Paul, MN USA; ^3^ HealthPartners Institute, Minneapolis, MN USA; ^4^ University of California San Diego, La Jolla, CA USA; ^5^ Queen’s University Belfast, Belfast, Northern Ireland United Kingdom

## Abstract

**Background:**

Cholinesterase inhibitors (ChEIs) are cornerstones in the symptomatic treatment of Alzheimer’s disease (AD) and dementia with Lewy bodies (DLB) and are also prescribed for vascular dementia (VD). Despite their widespread use, patterns of prescribing ChEIs are not fully understood.

**Objective:**

Examine the prevalence, timing, and types of ChEI prescriptions before and after dementia diagnosis including prescribing patterns by patient sex and race.

**Methods:**

We analyzed electronic health record and claims data for patients diagnosed with AD, DLB, or VD between October 2015 and August 2022 from a large healthcare system in the U.S. Claims for ChEI prescriptions (donepezil, galantamine, rivastigmine) were identified in the ±3 years surrounding dementia diagnosis. Repeated measures logistic regression was used to estimate the likelihood of ChEI prescription by time‐period, dementia type, and time x dementia type interaction to determine if change in prescription patterns significantly differed by diagnosis.

**Results:**

A total of 3166 eligible patients were identified (AD n = 2478; DLB n = 175; VD n = 513, Table 1). The number of patients with a ChEI prescription was higher in the post‐index period for all diagnosis groups. Patients with DLB had the highest prevalence of ChEI prescriptions both pre‐and post‐diagnosis (39% and 59%, respectively) compared to patients with AD (28% and 38%) and VD (13% and 14%; Table 2). For patients with a ChEI prescription post‐diagnosis, the first ChEI prescription was most likely to be donepezil (96%). When accounting for differences in age, sex, and race, patients with VD were found to have significantly lower rates of ChEI prescriptions compared to patients with DLB (OR: 0.47, 95% CI 0.3, 0.8). There was no difference in ChEI prescriptions observed between DLB and AD patients. In the fully adjusted model, females (OR: 0.81, 95% CI: 0.71, 0.91) and non‐white patients (OR: 0.74, 95% CI: 0.62, 0.88) were less likely to have a prescription for a ChEI (Table 3).

**Conclusion:**

Donepezil was the most frequently prescribed ChEI for AD, DLB, and VD. Patients with DLB had the highest prevalence of ChEI prescriptions both pre‐ and post‐diagnosis. Females and non‐white patients were less likely to receive ChEI prescriptions, underscoring potential disparities in treatment patterns.